# Injectable drug-loaded thermosensitive hydrogel delivery system for protecting retina ganglion cells in traumatic optic neuropathy

**DOI:** 10.1093/rb/rbae124

**Published:** 2024-10-24

**Authors:** Lei Wang, Yan Jiang, Yili Yao, Yudan Deng, Zhiqiang Liu, Jiangtao Ding, Wenwen Wang, Hao Chen, Kaihui Nan, Lingli Li

**Affiliations:** School of Ophthalmology & Optometry and Eye Hospital, Wenzhou Medical University, Wenzhou, Zhejiang 325027, China; State Key Laboratory of Ophthalmology, Optometry and Visual Science, Wenzhou Medical University, Wenzhou, Zhejiang 325027, China; Wenzhou Institute, University of Chinese Academy of Sciences, Wenzhou, Zhejiang 325000, China; The Affiliated Xiangshan Hospital, Wenzhou Medical University, Ningbo, Zhejiang 315700, China; School of Ophthalmology & Optometry and Eye Hospital, Wenzhou Medical University, Wenzhou, Zhejiang 325027, China; State Key Laboratory of Ophthalmology, Optometry and Visual Science, Wenzhou Medical University, Wenzhou, Zhejiang 325027, China; Refractive Surgery Center, Chongqing Eye and Vision Care Hospital, Chongqing 40042, China; School of Ophthalmology & Optometry and Eye Hospital, Wenzhou Medical University, Wenzhou, Zhejiang 325027, China; School of Ophthalmology & Optometry and Eye Hospital, Wenzhou Medical University, Wenzhou, Zhejiang 325027, China; School of Ophthalmology & Optometry and Eye Hospital, Wenzhou Medical University, Wenzhou, Zhejiang 325027, China; School of Ophthalmology & Optometry and Eye Hospital, Wenzhou Medical University, Wenzhou, Zhejiang 325027, China; Engineering Research Center of Clinical Functional Materials and Diagnosis & Treatment Devices of Zhejiang Province, Wenzhou Institute, University of Chinese Academy of Sciences, Wenzhou, Zhejiang 325000, China; School of Ophthalmology & Optometry and Eye Hospital, Wenzhou Medical University, Wenzhou, Zhejiang 325027, China; State Key Laboratory of Ophthalmology, Optometry and Visual Science, Wenzhou Medical University, Wenzhou, Zhejiang 325027, China; School of Ophthalmology & Optometry and Eye Hospital, Wenzhou Medical University, Wenzhou, Zhejiang 325027, China; State Key Laboratory of Ophthalmology, Optometry and Visual Science, Wenzhou Medical University, Wenzhou, Zhejiang 325027, China; School of Ophthalmology & Optometry and Eye Hospital, Wenzhou Medical University, Wenzhou, Zhejiang 325027, China; State Key Laboratory of Ophthalmology, Optometry and Visual Science, Wenzhou Medical University, Wenzhou, Zhejiang 325027, China; Wenzhou Institute, University of Chinese Academy of Sciences, Wenzhou, Zhejiang 325000, China; Zhejiang Engineering Research Center for Tissue Repair Materials, Wenzhou Institute, University of Chinese Academy of Sciences, Wenzhou, Zhejiang 325000, China

**Keywords:** TON, retinal ganglion cells, thermosensitive hydrogel, CNTF, TA

## Abstract

Currently, generalized therapy for traumatic optic neuropathy (TON) is lacking. Various strategies have been developed to protect and regenerate retinal ganglion cells (RGCs) after TON. Intravitreal injection of supplements has been approved as a promising approach, although serious concerns, such as low delivery efficacy and pain due to frequent injections, remain. In this study, we tested an injectable thermosensitive hydrogel drug delivery system engineered to deliver ciliary neurotrophic factor (CNTF) and triamcinolone acetonide (TA). The results of rheological studies showed that the prepared drug-loaded hydrogel possessed a suitable mechanical modulus of ∼300 Pa, consistent with that of vitreum. The hydrogel exhibited thermosensitive with sustained drug release performance. *In vitro* co-culture of the CNTF-loaded hydrogel system with primary RGCs also induced significant axon regeneration, with 38.5% increase in neurite length, indicating the regenerative response of the thermosensitive hydrogel drug delivery system. A Sprague-Dawley rat optic nerve crush model was constructed and applied to determine the neuroprotective and regenerative capacities of the system. The results demonstrated that a single intravitreal injection of the drug-loaded hydrogel (PLGA-PEG-PLGA + TA or PLGA-PEG-PLGA + CNTF) significantly increased RGC survival at both 14 and 28 days. The RGC survival rate was 31.05 ± 1.41% for the drug-loaded hydrogel system (the control group was 16.79 ± 1.50%) at Day 28. These findings suggest that the injectable drug-loaded thermosensitive hydrogel delivery system is a promising therapeutic tool for treating optic nerve degeneration.

## Graphical abstract

**Figure:**
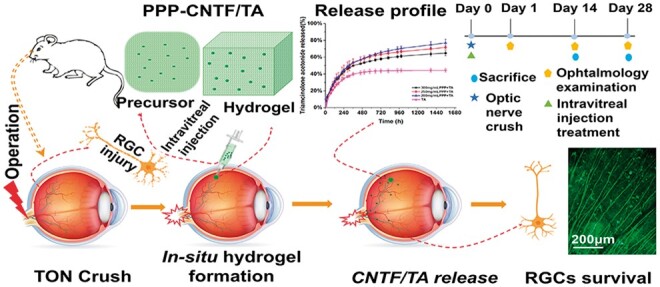

## Introduction

The optic nerve (ON) is a specialized sensory nerve that is responsible for vision. It connects the neural retina to the brain: the axons of the ON are extensions of the retinal ganglion cells (RGC) that form the nerve fibre layer of the neural retina. Traumatic optic neuropathy (TON) refers to a serious vision-threatening condition, in which acute injury to the optic nerve is caused by ocular or head trauma. The overall incidence of TON is 0.7–2.5%, with an average age of patients being 34 years [1]. With increased incidence, TON became a severe clinical disease with severity ranging from simple contusion to complete avulsion, leading to partial or permanent vision defect. TON is characterized by irreversible damage to the RGCs and progressive damage to the optic nerve [2]. However, the RGCs of adult mammals lose their axon regenerative capacity via elusive biochemical or molecular mechanisms [3]. Therefore, maintaining the viability of RGCs and promoting their axonal regeneration is key to optic nerve repair after TON. To date, established unified strategies for the treatment of optic nerve damage are lacking.

Clinically, intravenous administration of hormones and/or neurotrophic factors, optic canal decompression and combined investigation and treatment are currently used to preserve the activity and survival of RGCs and promote the regeneration of RGC axons [4, 5]. Compared to the surgical treatment of optic canal decompression, which may result in a series of post-operative complications (brain abscess due to protrusion of broken bone or local infection complicated by sinusitis), the administration of drugs provides considerably milder treatments. However, owing to their physiological and anatomical specificity, traditional intravenous and ocular surface administration of drugs is still associated with various concerns, such as frequent dosing, poor intraocular absorption, short duration of drug maintenance, ocular adverse reactions, systemic side effects and high surgical costs [6, 7]. In addition, the three therapies for managing TON do not differ significantly [8]. Consequently, continuous efforts have been made to improve the survival rate of RGCs and axonal regeneration after TON.

Intravitreal injection of therapeutic agents, an important treatment method for ophthalmic diseases (e.g. macular oedema [9], choroidal neovascularisation [10], retinal neovascularisation [11], neovascular glaucoma and intraocular infection [12]), has various advantages, such as the complications associated with the crossing of the blood–eye barrier are absent and targeted drug delivery becomes feasible, which actively enhances the therapeutic effect, reduces systemic toxicity and achieves better control of the disease. This treatment is hypothesized to protect the optic nerve, as the agents can efficiently reach the retina and positively affect RGCs. Ciliary neurotrophic factor (CNTF) promoted both the survival and regeneration of neurite extension of RGCs after TON *in vivo* in animal trials [13]. It has been shown to consistently protect the optic nerve by activating the Janus kinase/signal transducers and activators of the transcription signalling pathway [14]. However, the potential inflammation and vascular dysfunction associated with frequent intravitreal injection gives rise to secondary optic nerve damage. Therefore, counteracting inflammation is of paramount importance to alleviate the apoptotic process of RGCs in TON [15], which further reduces free radical damage and production of inflammatory and vasoactive factors, and moderates cellular homeostasis [16]. Triamcinolone acetonide (TA), a lipophilic steroid commonly used in ophthalmology, exhibits excellent anti-inflammatory properties [17]. It also prevents vasoconstriction and ensures blood supply to the optic nerve [18]. Therefore, intravitreal injection of CNTF/TA may be a new strategy for treating TON.

However, direct injection into the vitreous chamber cannot provide sustained delivery of CNTF and maintain high retention of TA for an extended period, whereas a constant supply is required for effective neuroprotection and axon regrowth after TON. Hence, researchers have developed numerous technologies to achieve long-term sustained delivery of effective concentrations of drugs [19, 20], including the use of polymeric micro/nanoparticles [21, 22], liposomes [23] and thermo-responsive hydrogels [24]. Among these treatments, injectable thermosensitive hydrogels have shown potential for the controlled release of water-soluble growth factors *in vivo* and compatibility with lipophilic agents, and have been considered as an attractive drug delivery system [25]. Polylactic-co-glycolic acid-polyethylene glycol-poly lactic-co-glycolic acid (PLGA-PEG-PLGA, PPP) is a triblock thermosensitive polymer that exhibits a flowable and injectable sol state at room temperature and a gel state at body temperature [26]. PPP has been ‘generally recognized as safe’ (G.R.A.S) with good biocompatibility by the Food and Drug Administration (FDA) [27–29]. It has been widely studied in biomedical applications recently because of its excellent capacity to encapsulate and deliver a wide range of hydrophilic and hydrophobic drugs [25, 26]. Moreover, for lipophilic drugs, PPP ensures better therapeutic effect due to improvement of solubility, dispersion, dissolution, low bioavailability and ability to impart *in vivo* stability to the lipophilic drugs [30]. Because of its polymeric nature, PPP hydrogels are proposed as suitable candidates for long-term intravitreal treatments [31].

In this study, we designed and prepared an injectable CNTF/TA-loaded PLGA-PEG-PLGA hydrogel and evaluated its RGC protection efficiency in TON. We investigated the physicochemical properties of the system and its efficacy to promote the survival and neurite extension of RGCs both *in vitro* and *in vivo*. This drug delivery system made full use of the amphiphilic structure of PPP to encapsulate different drugs and reconciled the advantages of intravitreal injection and may be used as a new biocompatible treatment for TON.

## Materials and methods

### Materials

PLGA-PEG-PLGA (PPP) was purchased from Shandong Jinan Daigang Biomaterial Co., Ltd (Shandong, China), and the average molecular weight was tested by gel permeation chromatography (GPC, TDA-305, Malvern, UK). TA was purchased from Shanghai Macklin Biochemical Technology Co., Ltd (Shanghai, China). Bovine serum albumin (BSA)-FITC and CNTF were purchased from Beijing Sinobio Company (Beijing, China). Primary rat RGCs were obtained from ProCell Life Science and Technology Co. Ltd (Wuhan, China). Human RPE and retinal Müller cells were from our laboratory. The 661 W photoreceptor cell line was provided by Shanghai Fuheng Biotechnology Co., LTD (Shanghai, China; No: FH0410). Healthy adult Sprague-Dawley (SD) female rats were purchased from the Laboratory Animal Resource Centre of Wenzhou Medical University (Shanghai, 2018-0004).

### Preparation of CNTF/TA-loaded thermosensitive hydrogel

Three different concentrations of the PPP solution, 300, 250 and 200 mg/ml, were prepared using phosphate-buffered saline (PBS). TA was suspended in a physiological saline solution (0.9%) to obtain a 400-mg/ml suspension. CNTF was dissolved in PBS to obtain a concentration of 1 mg/ml. The drug-loaded PPP hydrogels were prepared by mixing the PPP solution and drug stock solution at the volume ratio of 10:1 at 4 °C. BSA was used as an alternative model for investigating the *in vitro* release and rheology of the CNTF-loaded PPP hydrogel. The gelation process was evaluated using the inversion method [32].

### Rheological characterization

The rheology of PPP, PPP + BSA and PPP + TA hydrogels was characterized using a rheometer (AR-2000; TA Instruments, USA). The storage modulus (*G*′) and loss modulus (*G*″) of the hydrogels were measured in the oscillatory mode using a parallel plate configuration with diameter of 25 mm and gap of 300 μm. The measurement was performed at controlled temperature of 20 − 55 °C (ramp rate: 2 °C/min) using a thin layer of silicon oil to seal the samples and prevent water evaporation. The gelation temperature of the formulation was evaluated by monitoring the storage and loss moduli at a fixed oscillation frequency of 1 Hz and strain of 1%. All tests were repeated at least thrice.

### *In vitro* release profile

The *in vitro* release behaviour of TA from the hydrogel was studied using a dialysis method in 50 ml tubes, and the 30, 25 and 20% PPP hydrogels were used as references. The 100 µl hydrogels (300, 250 and 200 mg/ml) loaded with 10 µl TA (400 mg/ml) were placed into dialysis tubes with molecular weight cut-off of 1000 Da and then incubated at 37.5 °C for 10 min to ensure total gelation of materials. One hundred microlitres of PBS mixed with 10 µl TA was used as the TA control group. Next, 40 ml of prewarmed PBS was added to the tubes. At pre-determined time points (2, 4, 6, 8, 10, 12, 24, 72, 96, 120, 144, 168, 216, 240, 288, 312, 360, 456, 504, 576, 648, 720, 816, 936, 984, 1368 and 1560 h), 3 ml of the supernatant was extracted and replaced with the same volume of fresh prewarmed PBS at 37.5 °C. The amount of TA released was quantified using the ultra-performance liquid chromatography (UPLC) method. The following parameters were used for UPLC: chromatographic column, Shim-pack XR-ODS III 2.0 mm I.D. × 150 mm l, 2.2 μm; flowing phase: acetonitrile: water = 40:60 (v/v); flowing rate: 0.4 ml/min; detection wavelength: 240 nm; column temperature: 30 °C; injection volume: 10 µl. Each experiment was repeated thrice.

BSA was used as a hydrophilic large molecule model drug for studying the *in vitro* release behaviour. The FITC-labelled BSA (1 mg/ml) was loaded into PPP hydrogels (300, 250 and 200 mg/ml) at 10% (v/v) ratio. The mixtures were placed in dialysis tubes (molecular weight cut-off: 100 000 Da) that were placed into 15 ml tubes and then incubated at 37.5 °C for 10 min to guarantee total gelation of materials. Next, 4 ml of prewarmed PBS was added to the tubes. At pre-determined time points (1, 2, 3, 5, 6, 7, 8, 9, 10, 11, 12, 13, 14, 24, 29, 33, 35, 37, 48 and 60 h), 0.8 ml of the supernatant was extracted and replaced with the same volume of fresh prewarmed PBS at 37.5 °C. The amount of BSA-FITC in the release medium was measured using a plate reader (Varioskan LUX, Thermo Fisher, USA.) at the excitation wavelength of 493 nm and emission wavelength of 493 nm. Each experiment was repeated thrice.

### *In vitro* biocompatibility assay

#### Cell culture

Primary RGC, RPE and Müller cells were used to investigate the biological responses to the hydrogels. RPE, Müller cells and 661 W cells were grown in T-75 cell culture flasks (Falcon, Italy) in Dulbecco’s modified Eagle’s medium (DMEDM; Sigma, USA) supplemented with 10% foetal bovine serum (FBS) and 1% penicillin-streptomycin at 37 °C in the presence of 5% CO_2_. Primary RGCs were cultured in neurobasal-A/B27 medium containing 2% B-27.

#### In vitro biocompatibility assay of PPP hydrogels

The *in vitro* biocompatibility of the PPP hydrogels was evaluated using an extraction method described by the International Organization for Standardization (ISO 10993-5). The ratio of hydrogel to the extraction vehicle was 0.1 g/ml (ISO 10993-12). Briefly, the sterile PPP hydrogel solutions (300, 250, 200 mg/ml) were added to the tubes for gelation at 37.5 °C for 10 min, following which certain volume of prewarmed complete cell culture medium was added. After 24 h, the leached liquor was collected from different cells for the cytocompatibility study. RPE, Müller cells, primary RGCs and 661 W cells were used to evaluate cell viability in the thermosensitive hydrogels. Two hundred microlitres of the cell suspension (1 × 10^4^ cells/well) was incubated in the 96-well plate at 37 °C in the presence of 5% CO_2_. After 24 h, the medium was replaced with 250 μl extracts and cultured for pre-determined durations. Samples were incubated with 10% cell counting kit-8 (CCK-8; Beyotime Biotechnology, Shanghai, China) solution in the culture medium for 3 h in the dark. The optical densities (OD) were measured using an enzyme-linked immunosorbent assay plate reader (Varioskan LUX, Thermo Fisher, USA). Cells cultured in the normal medium were used as controls. Cell viability (%) was obtained as follows: OD_test_ as OD_control_ × 100%, where OD_test_ is the OD value of the test group and OD_control_ is the OD value of the control group. A live/dead assay (Life Technologies, Waltham, MA, USA) was used to estimate the viability of RPE, Müller cells and 661 W cells after co-culture with the extracts. Live cells stained green, and dead cells stained red. All the samples were incubated in live/dead dye solution (0.25 μl calcein-AM dye and 1.0 μl ethidium homodimer-1 dye in 0.5 ml of PBS) for 30 min. After incubation, the samples were washed thrice with PBS and observed under a DMi8 microscope (Leica, Germany). All experiments were performed in triplicates. Anti-β-III-tubulin staining was performed to evaluate the viability of RGCs after co-culture with the extractions.

#### Cytotoxicity of PPP + CNTF and PPP + TA hydrogels

The cytotoxicity of the PPP + TA hydrogel against RPE and that of the PPP CNTF hydrogel against RGC was investigated using the transwell method. Briefly, cells were seeded in a 24-well plate at the density of 3 × 10^5^ cells/ml for RPE and 2 × 10^5^ cells/ml for RGC. The cells were then incubated in a complete culture medium for 24 h. The drug-containing hydrogels containing different final concentrations of TA or CNTF were formed in the transwell chamber at 37 °C. The gelled drug-loaded hydrogel was then inserted into the cell-seeding plate with fresh medium (1.2 ml/well) and incubated for another 24 h before removing the transwell and measuring cell viability using the CCK-8 assay. Live/dead staining of RPE cells and anti-β-III-tubulin staining of RGCs were performed as mentioned in ‘Cell culture’ section. All experiments were performed in triplicates. Before RGC culture, the plate was pre-coated with polylysine (0.1 mg/ml) for 4 h.

### *In vitro* neurite extension of RGCs

Primary RGCs were used to investigate the *in vitro* neurite extension-promoting capacity of PPP + CNTF hydrogels. Briefly, cells (1 × 10^5^ cells/well) were seeded in a 24-well plate pre-coated with 0.1 mg/ml polylysine. After culturing for 24 h, the medium was replaced with CNTF-containing culture medium and PPP + CNTF hydrogels in the transwell chambers. Pure CNTF-containing medium (0.05, 0.1, 0.2, 0.4 μg/ml) was used as the control. The concentration of CNTF in the PPP hydrogels (300 mg/ml) was set to 0.4, 0.8 and 1.6 μg/ml. Immunofluorescence staining of the neuron-specific marker, β-III-tubulin, was used to determine the neurite length of RGCs. Ten images were captured randomly from each well in each group. The growth of RGC axons was analyzed using the ImageJ software (Java 1.8.0_112, National Institute of Health, USA).

### *In vitro* H_2_O_2_-induced oxidative stress model

To test the dose-dependent oxidative stress induced by H_2_O_2_, 661 W cells were treated with different concentrations of H_2_O_2_ (200–600 μM) for 48 h. The appropriate dose of H_2_O_2_ that provided the optimum cytotoxicity or oxidative stress was then selected. To assess the cytoprotective effect of CNTF against H_2_O_2_-mediated cell death, 661 W cells were subjected to a co-treatment plan of 48 h H_2_O_2_ and CNTF-containing culture medium. Flow cytometry was employed to evaluate the reactive oxygen species (ROS) production using 2′-7′-dichlorofluorescein diacetate (DCFH-DA, Sigma-Aldrich, #D6883). The highly fluorescent compound 2′-7′-dichlorofluorescein (DCF) is formed when ROS oxidized nonspecific esterase-cleaved DCFH-DA. The 661 W cells were incubated with 5 µM DCFH-DA at 37 °C for 20 min, followed by washing the cells twice with PBS. Intracellular H_2_O_2_ or low molecular-weight peroxides oxidize DCFH-DA to the highly fluorescent compound DCF. Signals were detected with a 525 nm bandpass filter (FITC) on an LSR II flow cytometer (Becton Dickinson, San Jose, CA, USA) and observed under a DMi8 microscope (LEICA, Germany).

### *In vivo* optic nerve crush model in rats and intravitreal injection therapy

A TON model was established to further verify the ability to protect RGC viability and promote neurite elongation *in vivo*. All the *in vivo* experiments were performed according to international regulations and laws on the protection of animals used for scientific purposes and the Chinese government (GB14925-2010). This study was approved by the Ethics Committee of Wenzhou Medical University (process code wydw2019-0912). Eighty rats (female, 6-month-old, weighing 200 − 250 g), procured from Jiesijie Experimental Animal Co. (Shanghai, China), were randomly divided into eight groups with 10 rats for each group: healthy (no treatment), control (TON without any treatment), NS (TON, intravitreal injection of 5 μl normal saline), PPP (TON, intravitreal injection of 5 μl PPP to a concentration of 300 mg/ml), TA (TON, intravitreal injection of 5 μl TA to a concentration of 40 mg/ml), PPP + TA (TON, intravitreal injection of 5 μl PPP + TA to obtain PPP 300 mg/ml and TA content 0.2 mg), CNTF (TON, intravitreal injection of 5 μl CNTF to a concentration of 0.1 mg/ml), PPP + CNTF (TON, intravitreal injection of 5 μl PPP + CNTF with the concentration of PPP being 300 mg/ml and that of CNTF being 0.5 μg). In the following paragraphs, the experimental group refers to all the groups except healthy group, the treated group refers to all the experimental group except control group. The rats were anaesthetized via peritoneal administration of xylazine and pentobarbital sodium (30 mg/kg). Proparacaine hydrate (0.5%) was applied to the surgical eye under local anaesthesia. The surgical procedure was as follows: (i) the area around the lateral corner of the eye was depilated and sterilized with iodophors, and the eye was irrigated using normal saline; (ii) a 5-mm incision (which can heal itself without suturing) was made on the skin around the lateral obit with scissors to partially expose the optic nerve; (iii) approximately 2 mm behind the eye globe, a pressure constant artery clamp (pressure ¼ 100 g, 19–6 mm × 1 mm) was used to crush the optic nerve for 10 s without damaging the surrounding vascular supply; (iv) the bulbar conjunctival sac was coated with ofloxacin to prevent postsurgical infection; (v) intravitreal injection was used to treat TON. The procedure was as follows. First, a 30-gauge insulin syringe was used to make an incision in the temporal pars plana of the surgical eye, approximately 1 mm beyond the limbus. Then, a Hamilton microsyringe (# 33-gauge, 10 mm) was inserted into the incision at a 45-degree angle to extract 5 μl of the contents, followed by injection of 5 μl of the therapeutic substance. After surgery, all animals were returned to their pre-marked cages and observed at pre-determined time points.

### *In vivo* ophthalmic observation

Slit-lamp microscopy was used to assess general abnormalities and inflammatory reactions, and optical coherence tomography (OCT) and electroretinography (ERG) were used to record retinal images for further structural and functional analysis. Before OCT observation (30D lens was inserted), all animals were anaesthetised via peritoneal administration of xylazine and pentobarbital sodium (30 mg/kg), and the surgical eyes were topically anaesthetised using 0.5% proparacaine hydrochloride eye drops and topicamide eye drops as mydriatics. Images were captured around the optic nerve head, and the Heidelberg analysis system was used to evaluate retinal thickness, including the thickness of the retinal nerve fibre layer (RNFL), ganglion cell complex (GCC) layer, (nerve fibre layer (NFL), ganglion cell layer (GCL), inner plexiform layer (IPL)) and the full thickness of the retina.

For the ERG photo-stress test, the animals were adapted to a dark environment 24 h before the test. After topical anaesthesia and application of mydriatics, a 2.5% methyl cellulose gel (an ionic conductive gel to improve the contact of electrodes with the cornea) was spread on the ocular surface for better and more reliable observation. The recording was performed binocularly according to the standards of the International Society for Clinical Electrophysiology of Vision (ISCEV) at 1, 14 and 28 days in the dark-adopted 0.01 ERG, dark-adopted 3.0 ERG, dark-adopted 3.0 ERG oscillatory potentials (Ops), light-adopted 3.0 potentials and light-adopted 30 Hz flicker. The rats were kept warm using a 37 °C warm mat during the whole test.

For the flash visual evoked potential (f-VEP) test, the rats were dark-adapted overnight and anaesthetized using isoflurane. Local anaesthesia was induced by a topical application of an eye drop (Benoxil ophthalmic solution 0.4%; Santen Pharmaceutical Co., Ltd), and the pupil was dilated with a 1:10 dilution of tropicamide (Santen Pharmaceutical Co., Ltd). The reference and ground electrodes were placed on the cheek and tail, respectively. The stimulus intensity was set at 3.00 cd*·*s/m^2^, with stimulus intensities (0.01, 3.00 and 10.0 cd*·*s/m^2^) selected according to the standard method recommended by the International Society of Clinical Electrophysiology of Vision (ISCEV). The high- and low-path filters were set to 1 and 500 Hz, respectively. The VEP responses were measured consecutively 100 times, and the waveforms were averaged.

### Retinal immunofluorescence staining

The rats were euthanized and the eyeballs were removed immediately and fixed in 4% paraformaldehyde at 4 °C for 30 min 14 and 28 days after TON and respective treatments. The retinas were carefully isolated under a stereoscopic microscope (Carl Zeiss) and four-leaf clover-shaped flat-mounted on a 48-well TCP. After rinsing thrice with PBS, the retinas were blocked using 10% BSA in PBS containing 0.2% Triton X-100 for 1 h at room temperature. The tissues were incubated in an anti-β-III-tubulin solution (1:300, Abcam) overnight at 4 °C. The tissues were washed thrice with 2% BSA in PBS containing 0.2% Triton X-100 (30 min each) and PBS for 10 min. The tissues were then incubated with goat anti-rabbit IgG H & L solution (1:200; Abcam) for 2 h at room temperature in the dark. The samples were washed thrice with PBS for 5 min each, followed by the addition of fresh PBS, and incubated in the dark before being photographed under a DMi8 fluorescence microscope (Leica, Germany). Eight images of each sample were captured at the centre and edge of the four-leaf clover of the retina. ImageJ (Java 1.8.8_112, National Institute of Health, USA) was used to analyse the survival rate of RGC. The RGC number was counted, and the RGC viability was calculated according to the equation: RGCs viability (%) = *N*_test_/*N*_healthy_ × 100%, where *N*_test_ is the RGC number of the test group and *N*_Healthy_ is the RGC number of the healthy group.

### Histopathological and immunohistochemical staining of the optic nerve

The optic nerves were carefully isolated and fixed in 4% paraformaldehyde at 4 °C for 30 min. The tissues were dehydrated in a graded series of ethanol of different concentrations and placed in two separate pots of xylene for 15 and 30 min. The optic nerves were embedded in paraffin wax for 1.5 h, with the longitudinal section facing upward for histopathological and immunohistochemical staining, and frozen sections were used for immunofluorescence staining. The tissues were sectioned at a thickness of 4 μm and spread in 50 °C water and salvaged using a glass carrier, followed by baking at 60 °C for 12 h. The slices were dewaxed twice in xylene and rehydrated using an ethanol gradient. After washing with distilled water, the sections were stained with haematoxylin and eosin (H&E) according to a standard protocol. Growth-associated protein-43 (GAP-43) is considered a marker of axonal regeneration. Immunohistochemical analysis was performed to quantify RGC survival. Hydrogen peroxide (3%), citrate repair solution (pH 6.0), and BSA were used to block endogenous peroxidases and non-specific adsorption. The GAP-43 primary antibody (1:600; Abcam) was added to the slices and incubated overnight at 4 °C. After washing thrice with PBS, biotinylated secondary antibodies (REAL EnVision HRP, DAKO, Copenhagen, Denmark) were added to the sections and incubated for 30 min at 37 °C. Diaminobenzidine (DAB, Phygene, Fuzhou, China) was added and incubated for 3–10 min for colour development. Haematoxylin was used for re-staining. After dehydration in alcohol gradient and treating with xylene for achieving transparency, the sections were mounted with neutral gum. At least 10 images from each sample were randomly selected for statistical analysis.

For immunofluorescence staining, the sections were incubated with the primary rabbit anti-IL-1β (1:500, Protein tech, RRID: 26048-1-AP) at 4 °C overnight, followed by the corresponding secondary antibodies Alexa Fluor^®^ 488 (1:500; Abcam) under room temperature for 2 h. The images were recorded using a confocal laser scanning microscope (CLSM, LSM880, Carl Zeiss Jena, Germany).

### Statistical analysis

Data analysis was performed using Prism7 software (GraphPad, San Diego, California, USA). All results are expressed as mean ± standard deviation, and analysis of variance (ANOVA) was used to determine significance among groups. *P* < 0.05 was considered statistically significant.

## Results

### CNTF or TA-loaded thermosensitive hydrogel preparation and rheological characterization

**Figure 1.**
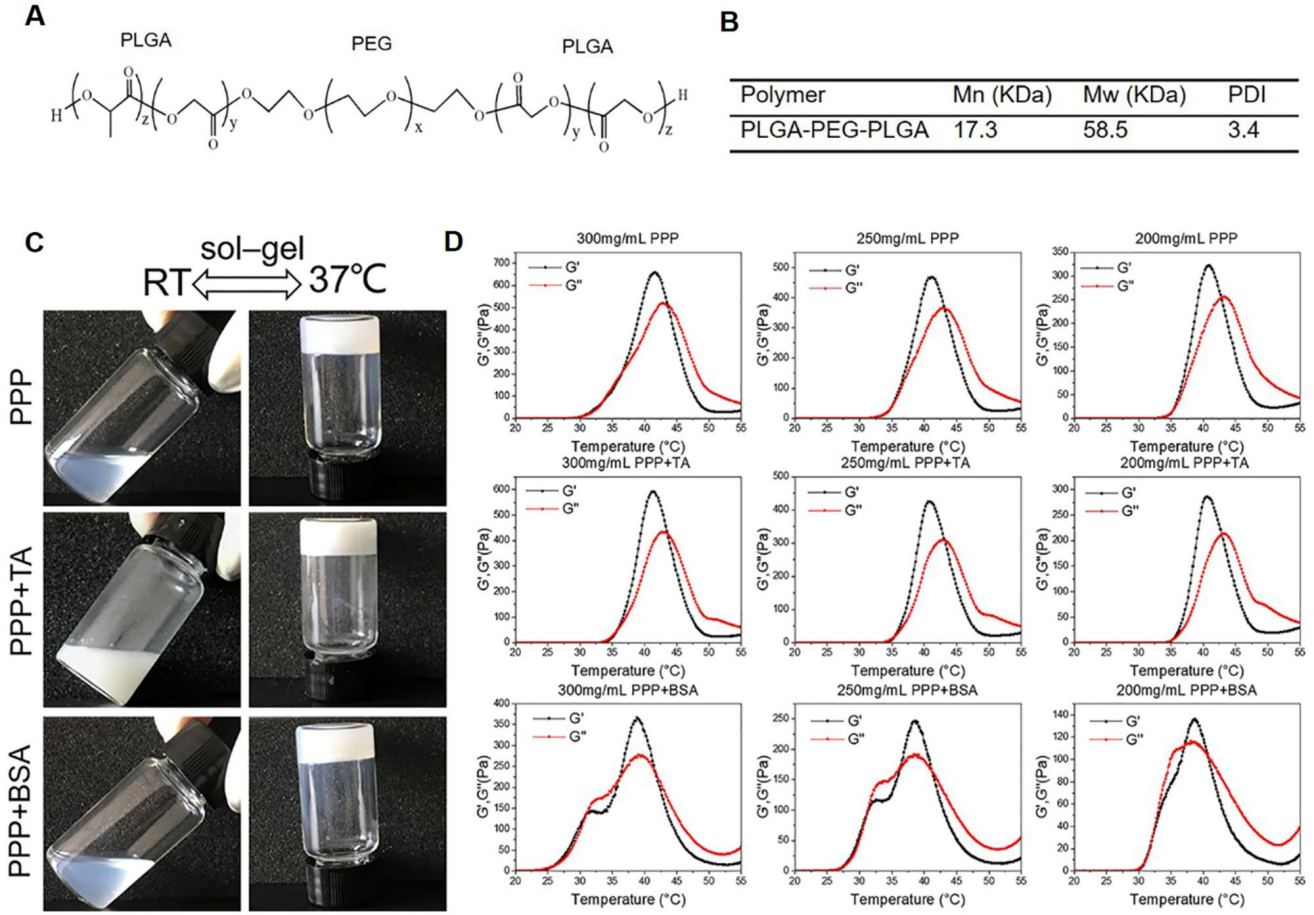
(**A**) The chemical structure of PLGA-PEG-PLGA. (**B**) Number average Mn and weight average Mw obtained by GPC. (**C**) Photographs of the temperature-sensitive hydrogel formation confirmed using the tube inversion method. (**D**) Rheological characterizations of the PPP hydrogels of 300, 250 and 200 mg/ml with TA and BSA. Temperature: 20–55 °C with a ramp rate of 2°C/min, oscillation frequency: 1 Hz, strain: 1%, plate diameter: 25 mm, gap: 300 μm.

Polylactic-co-glycolic acid-polyethylene glycol-poly lactic-co-glycolic acid (PLGA-PEG-PLGA, PPP) is a triblock polymer as shown in Figure 1A. The average molecular weight was tested to be 58.5k Da (Figure 1B). Thermosensitive PPP hydrogel formation was confirmed using the tube inversion method with a criterion of flow or non-flow over 1 min [32]. The representative images are shown in Figure 1C. Each individual copolymer solution prepared as 30%, 25%, or 20% suspension in PBS (0.01 M, pH 7.4) showed a sol-gel transition within 1 − 2 min when the environmental temperature changed from room temperature to 37.5 °C. This property was further confirmed via rheological characterization, as shown in Figure 1D. The transition temperature ranges of the 300, 250 and 200 mg/ml PPP hydrogel were 36.6 − 43.7, 35.2 − 43.2 and 35.2 − 42.9 °C, respectively. All three hydrogel formulations maintained their hydrogel structures at body temperature. The transition temperatures of PPP + BSA and PPP + TA were the same as that of the PPP hydrogel, showing stable gel state between 35 °C and 40 °C. The storage modulus (*G*′) and loss modulus (*G*″) of the PPP, PPP + TA and PPP + BSA hydrogel at body temperature (37.5 °C) is shown in Table 1. The *G*′ of the 300, 250 and 200 mg/ml PPP hydrogels was 293, 200 and 114 Pa, respectively. After loading with TA, *G*′ increased for the 300 mg/ml group to 553 Pa and decreased for the 250 mg/ml (136 Pa) and 200 mg/ml (88 Pa) groups. The *G*′ of the PPP + BSA hydrogels increased in all the 300 mg/ml (322 Pa), 250 mg/ml (228 Pa) and 200 mg/ml (124 Pa) groups.

**Table 1.** The storage modulus (*G*′) and loss modulus (*G*″) of the sample at 37.5 °C

	300 mg/ml PPP	300 mg/ml PPP+TA	300 mg/ml PPP+BSA	250 mg/ml PPP	250 mg/ml PPP+TA	250 mg/ml PPP+BSA	200 mg/ml PPP	200 mg/ml PPP+TA	200 mg/ml PPP+BSA
*G*′ (Pa)	293	553	322	200	136	228	114	88	124
*G*″ (Pa)	255	426	254	158	79	186	81	43	114

### *In vitro* drug release

**Figure 2.**
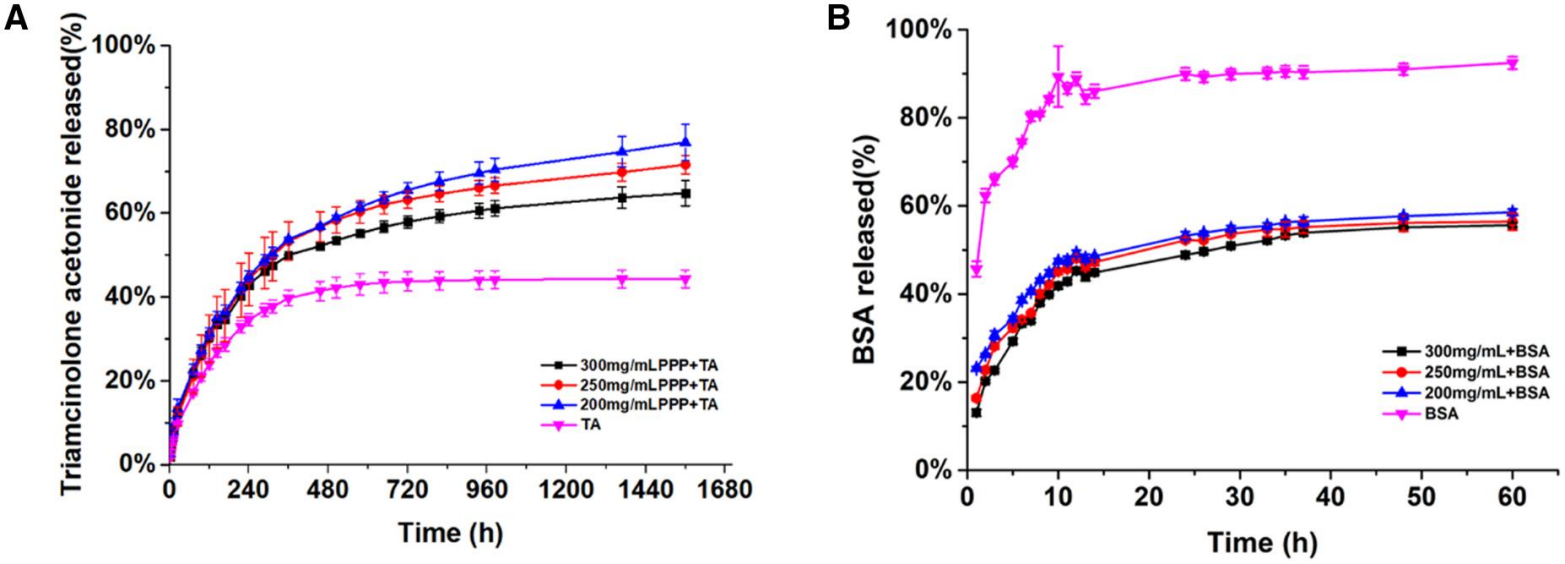
*In vitro* cumulative release profiles of TA (**A**) and BSA (**B**).

TA was used to investigate the *in vitro* hydrophobic drug release kinetics of the PPP hydrogels, which were analyzed at 37 °C in PBS and compared to that of TA alone as the control. The percentages of TA released were calculated with respect to time, and the results shown in Figure 2A exhibit a two-stage release pattern, with fast release at different percentages in the first 10 days, followed by a sustained-release phase until the end of the release study. The release percentages of TA in the first 10 days were ranked in the order of 200 mg/ml > 250 mg/ml > 300 mg/ml. The rate of TA released from the PPP hydrogel was significantly higher than that from the control group. After 24 days, the cumulative release of the TA control group reached a plateau. In contrast, the cumulative release of TA from the PPP hydrogels followed a controlled step-by-step pattern, and the release percentages were 55.21 ± 0.91, 60.38 ± 2.69 and 61.48 ± 1.06% for 300, 250 and 200 mg/ml, respectively, which was significantly higher than that of the TA control group (42.99% ± 2.57%). After 65 days, the cumulative release of TA was 64.79 ± 3.03% for the 300 mg/ml PPP hydrogel group, which was significantly lower than that of the 250 and 200 mg/ml PPP hydrogel groups. This result is consistent with previous studies [33].

BSA was chosen as a model drug to evaluate the *in vitro* hydrophilic drug release [34], as shown in Figure 2B. A two-stage release pattern was detected in all hydrogels, with an initial rapid release in the first 14 h, followed by a sustained release. The BSA control group showed the most rapid release with a cumulative release percentage of 89.95 ± 1.39% in the first 14 h. The PPP hydrogel groups exhibited sustained release during the subsequent hours. At 37 h, the cumulative release percentages were 53.90 ± 0.59, 55.20 ± 1.40 and 56.50 ± 1.01% for the 300, 250 and 200 mg/ml PPP hydrogel groups, respectively.

### *In vitro* biocompatibility

**Figure 3.**
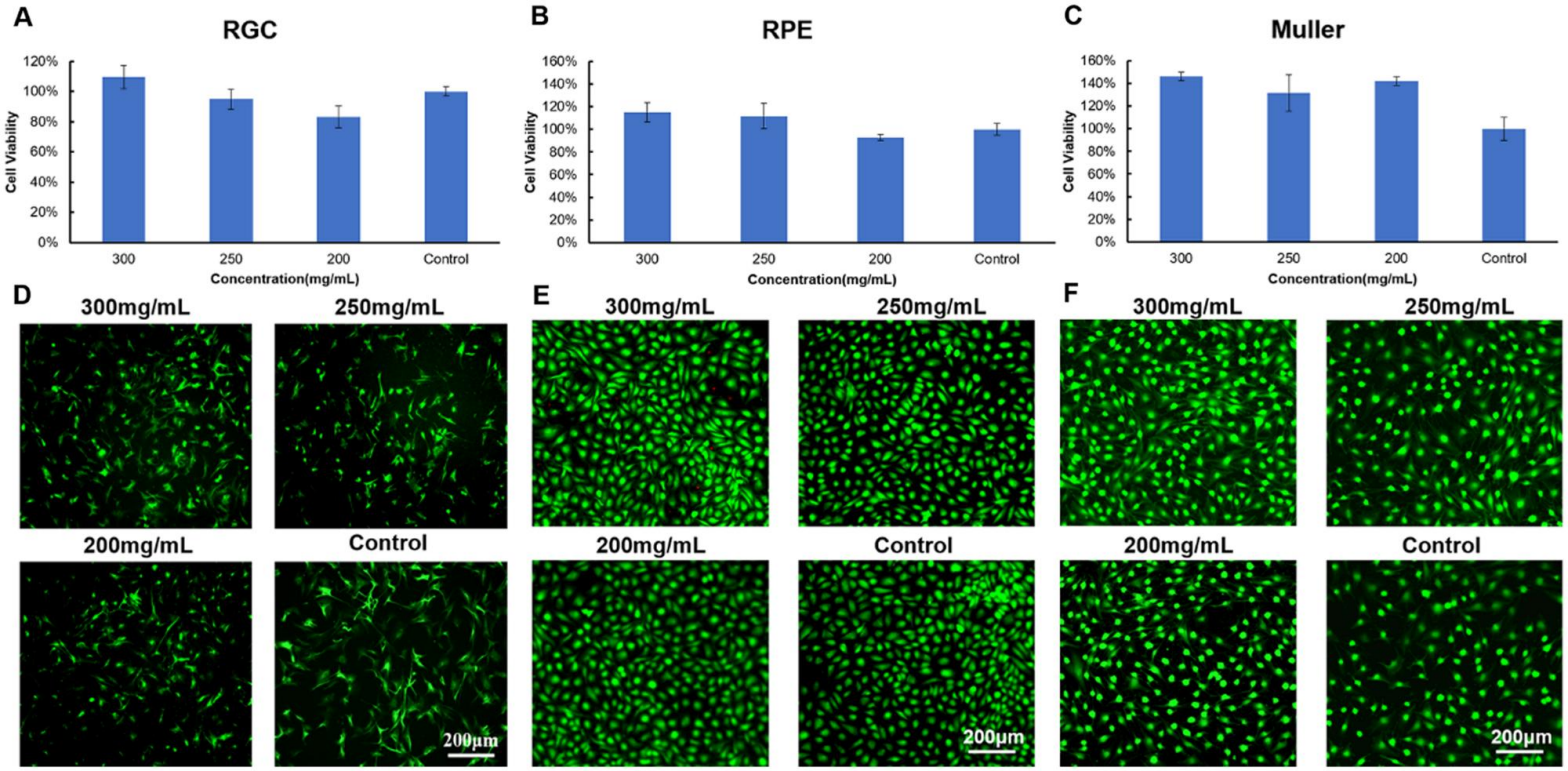
*In vitro* biocompatibility of the 300, 250 and 200 mg/ml PPP hydrogels. (**A**) RGC cell viability; (**B**) RPE cell viability; (**C**) Müller cell viability; (**D**) anti-β-III-tubulin staining of RGCs after 1 day of culture; (**E** and **F**) live/dead staining of RPE and Müller cells after 1 day of culture. Bar: 200 μm.

**Figure 4.**
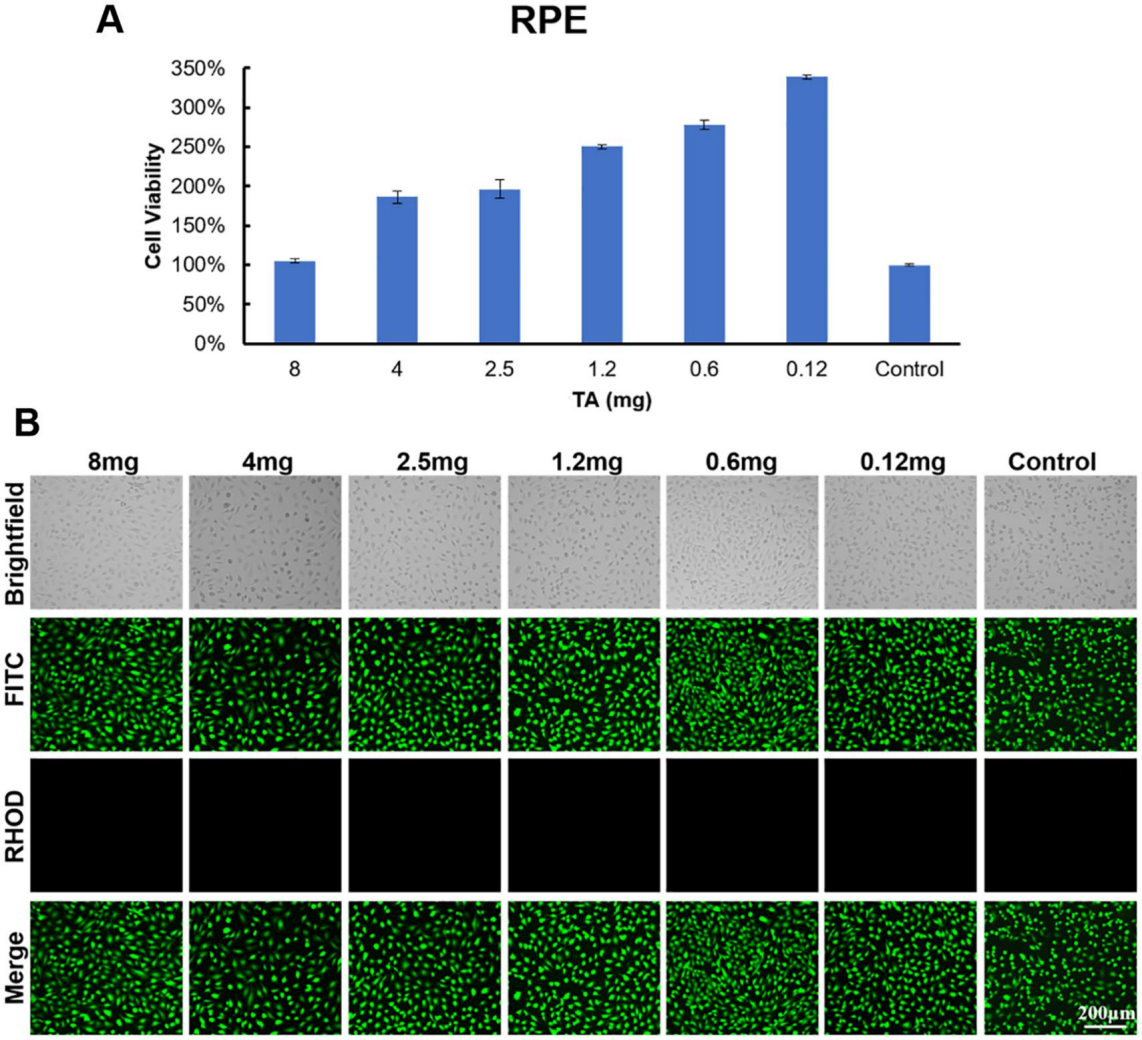
*In vitro* biocompatibility of the 300 mg/ml PPP hydrogels loaded with various concentrations of TA after 1 day of culture. (**A**) RPE cell viability; (**B**) representative live/dead fluorescence images of RPE cells cocultured with PPP + TA hydrogels. Bar: 200 μm.

**Figure 5.**
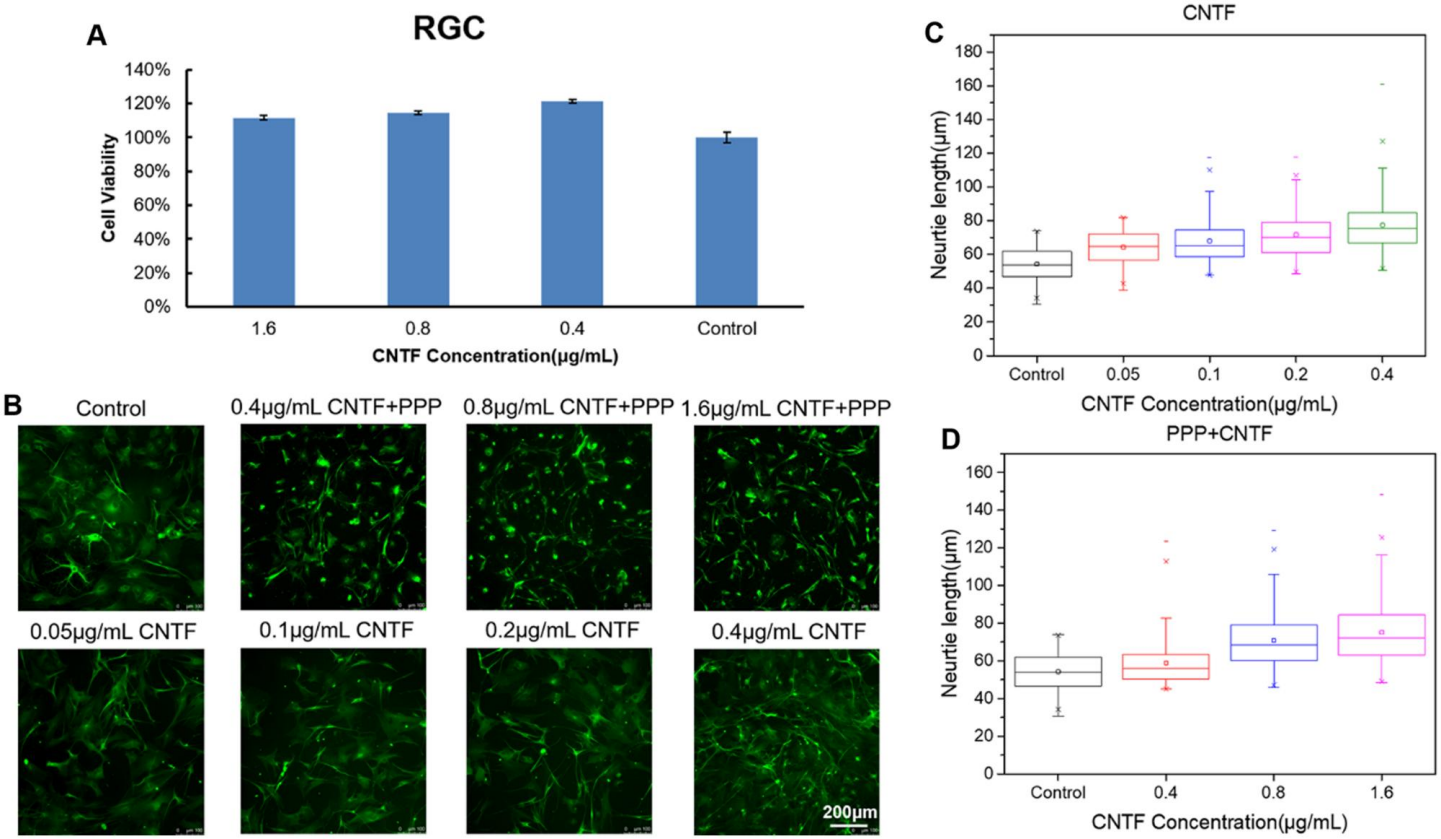
(**A**) *In vitro* biocompatibility of the 300 mg/ml PPP hydrogels loaded with various concentrations of CNTF after 1 day of culture. (**B**–**D**) *In vitro* neurite extension of the primary RGCs cocultured with PPP + CNTF hydrogels after 3 days of culture. (**B**) Representative images of the RGCs stained using anti-β-III-tubulin as a specific biomarker. (**C**) Quantitation of neurite outgrowth of RGCs in the presence of various CNTF. (**D**) Quantitation of neurite outgrowth of RGCs in the presence of PPP + CNTF hydrogels. Bar: 200 μm.

RGC, RPE, Müller cells and 661 W cells were used to evaluate the *in vitro* biocompatibility of the PPP hydrogel according to ISO 10993-5. The viability of retinal cells cultured with the extract of PPP hydrogels with solid contents of 300, 250 and 200 mg/ml was higher than 80% as shown in Figure 3A–C. The results of the live/dead staining assay (Figure 3E and F) showed that the RPE and Müller cells were green, while no cells showed red fluorescence for any of the PPP hydrogels. The cells were uniformly attached to the culture plate; they showed outstretched morphology and the density of live cells increased with culture time. The results of anti-β-III-tubulin staining (Figure 3D) showed that all the RGC were active and exhibited a uniform spreading morphology in all the PPP hydrogel groups. Thereafter, we investigated the *in vitro* biocompatibility of the PPP hydrogels (300 mg/ml) loaded with TA (Figure 4) and CNTF (Figure 5A). The viability of the RPE cells exceeded 100% in the PPP + TA hydrogel groups treated with various concentrations of TA. The results of live/dead staining showed that all cells were green, and no cells showed red fluorescence for any of the PPP + TA hydrogel groups after co-culture for 24 h. The cells were well adhered to the culture plate with an outstretched morphology. As shown in Figure 5A, the viabilities of RGCs in the PPP + CNTF hydrogel groups exceeded 100% after 24 h, with no significant difference between various concentrations of CNTF. The results of β-III-tubulin staining also showed that the RGCs were active after 3 days (Figure 5B).

### Assessment of neurite length *in vitro*

Primary RGCs were used to investigate the *in vitro* neurite extension-promoting capacity of PPP + CNTF hydrogels. Three days after treatment, 0.05, 0.1, 0.2 and 0.4 μg/ml CNTF were found to be associated with significantly more neurite growth than the control (Figure 5C, P* *<* *0.05). The neurite length increased with the concentration of CNTF. In this study, the 300 mg/ml PPP hydrogel was selected for assessment. PPP + CNTF (0.4, 0.8 and 1.6 μg/ml final concentration of CNTF) was used to investigate the effect on neurite length *in vitro*, as shown in Figure 5B and D. The neurite length of primary RGCs in the PPP + CNTF group was significantly higher than that in the control group (*P *<* *0.05). The promotional effect of the PPP + CNTF hydrogel was also concentration-dependent. In addition, the cell bodies of primary RGCs in the PPP + CNTF group were smaller than those in the control and CNTF groups.

### Assessment of the toxicity of the hydrogels *in vivo* via intravitreal injection

**Figure 6.**
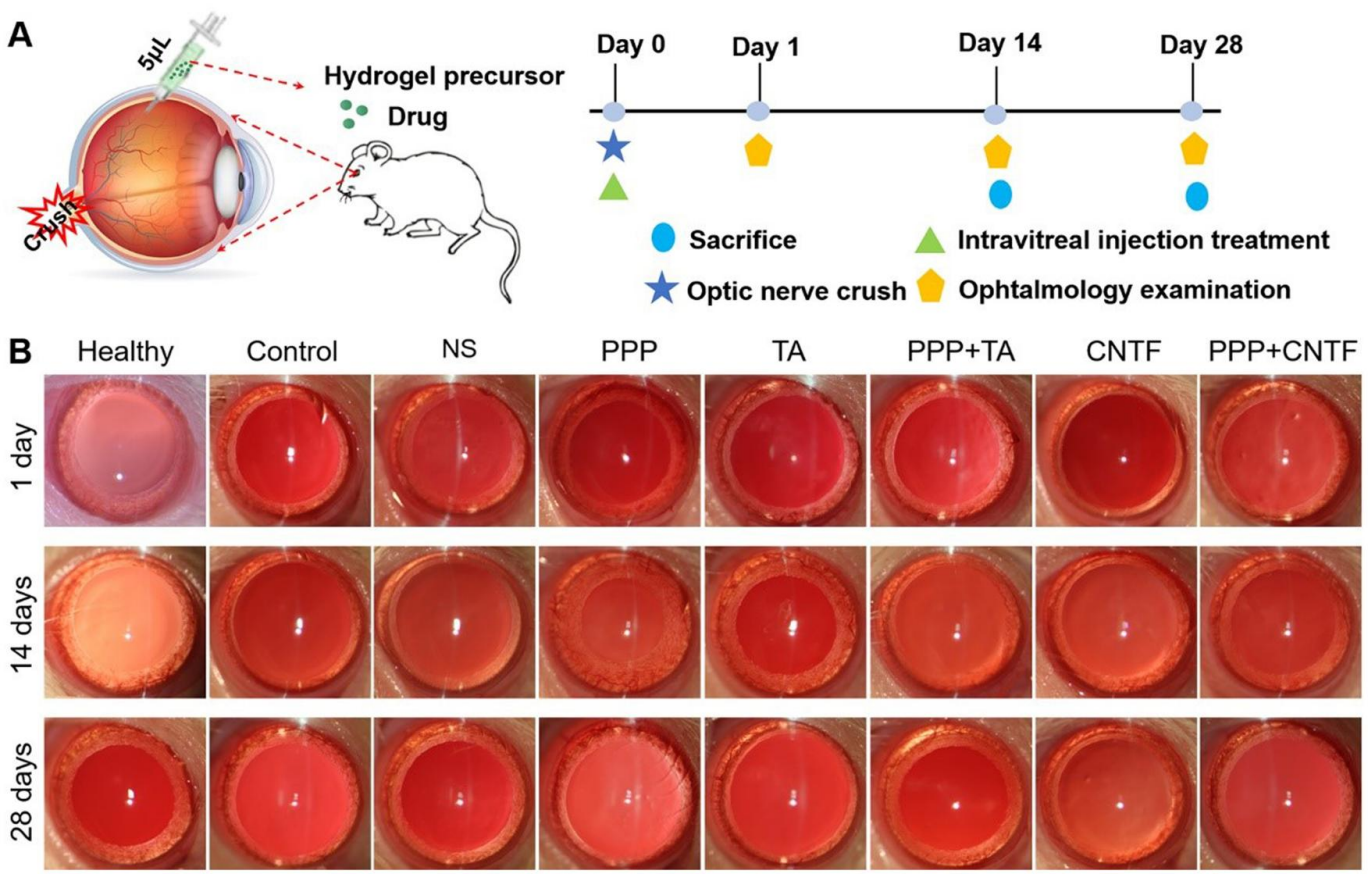
(**A**) Schematic diagram showing the construction of the *in vivo* optic nerve crush model and intravitreal injection treatment, and the time schedule of the treatment; (**B**) representative slit-lamp images of the anterior chamber in each group after 1, 14 and 28 days of treatment.

All the hydrogels were administered via intravitreal injection in a minimally invasive manner. The hydrogel precursors underwent a sol-to-gel phase transition and formed a stable hydrogel state *in situ* after injecting into the vitreous cavity, as illustrated in Figure 6. The *in vivo* effectiveness of the drug-loaded hydrogels was assessed using the TON model. Figure 6B shows representative slit-lamp images of the anterior chamber after 1, 14 and 28 days of treatment. Compared with the control groups, the hydrogel groups (PPP + TA, PPP + CNTF and PPP) appeared considerably whiter from an in-depth view, indicating that the hydrogels were still stable *in vivo* 28 days post-treatment. In addition, in all hydrogel groups, the corneas were transparent without oedema, the conjunctiva was the same as that in the healthy group without hyperaemia, and negligible inflammation was observed in any hydrogel group. These results indicated that the hydrogel possessed excellent *in vivo* biocompatibility.

### Evaluation of retinal function *in vivo*

**Figure 7.**
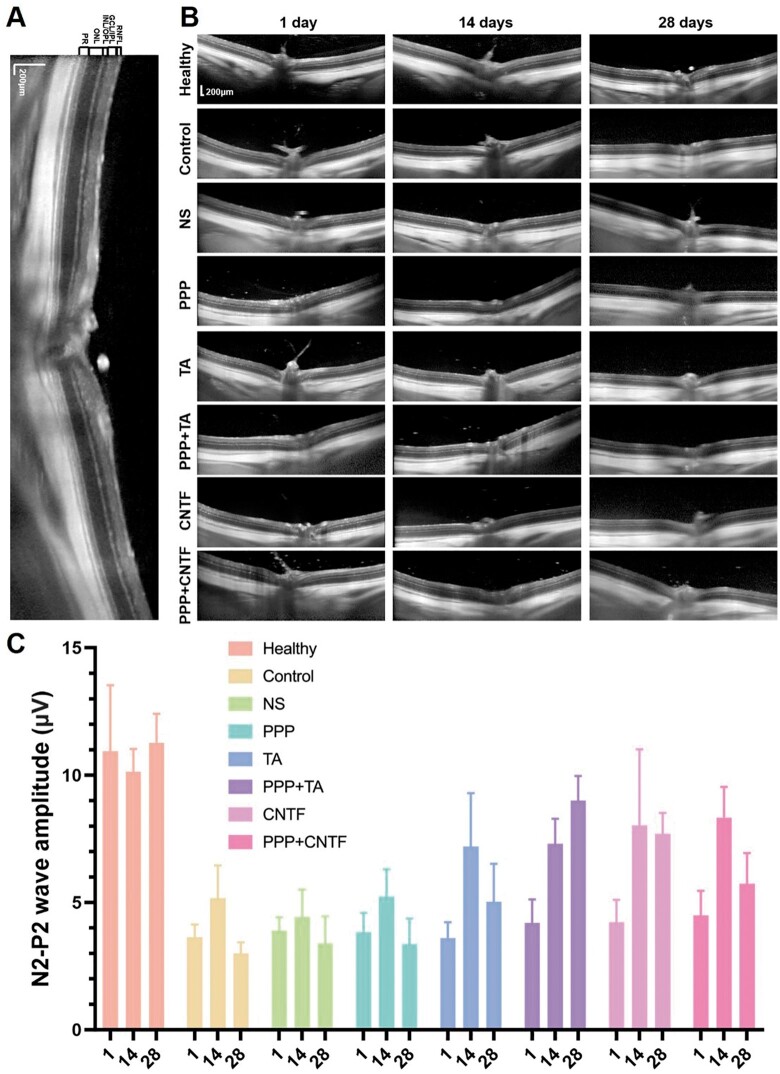
(**A**) Representative magnified OCT images of healthy retinas; (**B**) representative OCT images of the retina structure of the various groups; (**C**) f-VEP analysis after 1, 14 and 28 days of treatment.

**Figure 8.**
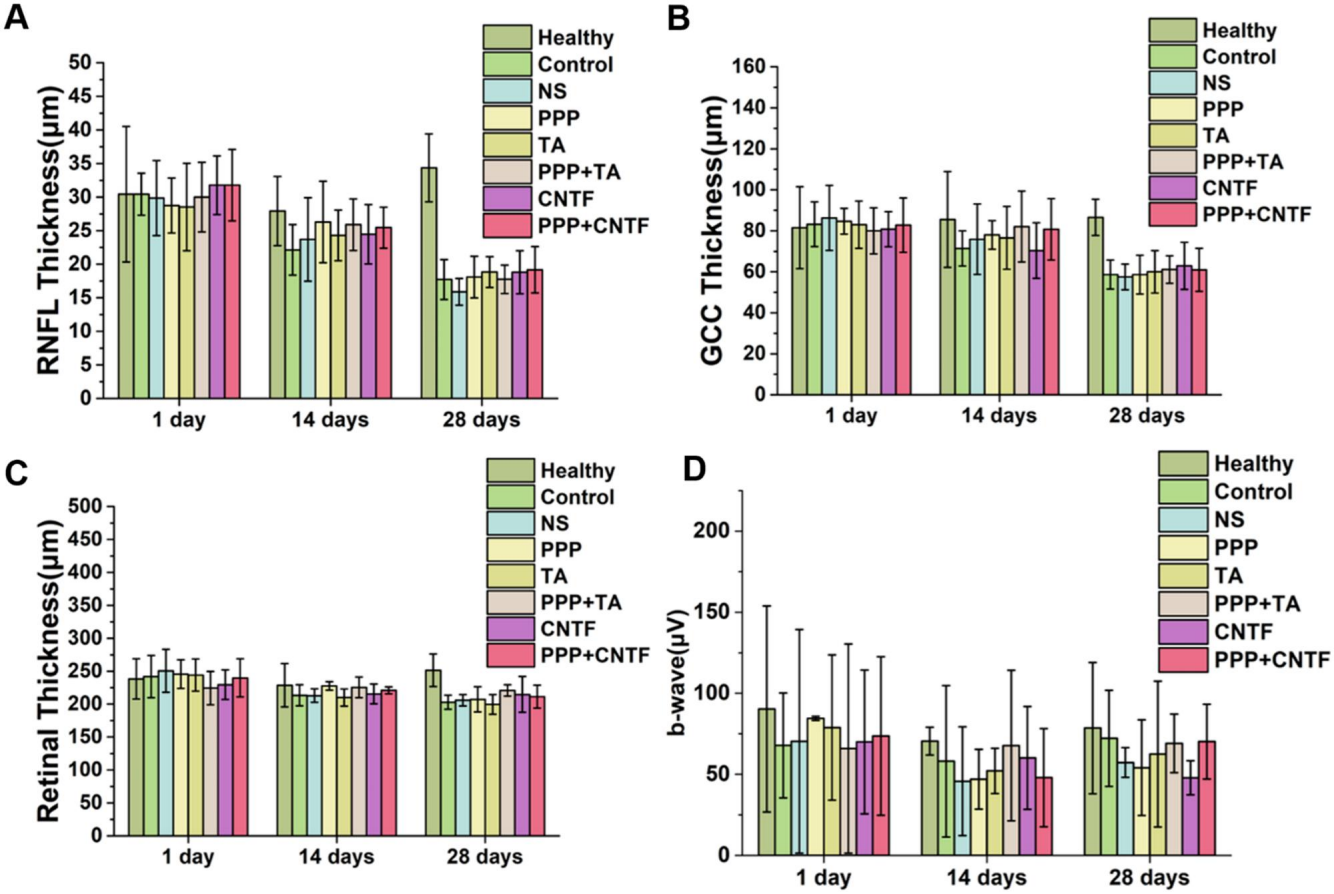
(**A**) RNFL thickness, (**B**) GCC thickness and (**C**) retinal thickness of the various groups after 1, 14 and 28 days of treatment. (**D**) The dark-adapted electroretinogram (ERG) recorded b-wave of the various groups after 1, 14 and 28 days of treatment.

Dilation of the pupils and disappearance of light reflection were observed in the control group animals (Figure 6B), and the blood supply to the fundus was normal, indicating the successful construction of the TON model. After TON and intravitreal injection, *in vivo* observation using OCT and ERG was performed to assess the structure and function of the retina at pre-determined time points, as shown in Figures 7 and 8A–C (OCT) and Figure 8D (ERG), respectively.

To better observe the multilayered structure of the retina, the RNFL, GCL, IPL, inner nuclear layer (INL), outer plexiform layer (OPL), outer nuclear layer (ONL) and photoreceptor cell layer (PR) were observed using an amplified image of a healthy retina (Figure 7A). The layered structure of the retina was also clearly observed in all the treated groups, as shown in Figure 7B. The retinal structure did not differ significantly between the treatment and control groups at 1 and 14 days. The thicknesses of the RNFL, GCC, and retina were analyzed quantitatively based on the OCT images (Figure 8A–C). The RNFL thickness was approximately 30 μm at 1 day and 25 μm at 14 days for all the groups without any significant differences. The GCC thickness was between 70  and 80 μm and did not differ significantly at 1 and 14 days among the groups. The retinal thickness in all the groups was approximately 230 μm, which did not change significantly at 1 and 14 days (all the retinal thicknesses were recorded at the same site, which was 500 μm away from the centre of the optic disc). The thicknesses of the RNFL, GCC and retina at 28 days were significantly lower than that at 1 and 14 days, however, there was no obvious difference among treated groups and control and NS groups. All the results suggested that the intravitreal injection of the thermosensitive hydrogels was biosafe, the retinal structure could be preserved in 14 days, and the RGC number in the retinal layer decreased after 28 days.

Dark-adapted ERG was used to evaluate the reflection of the internal and bipolar neurons of the retina (the main function of the retina) by measuring the amplitude changes of the b-wave (the distance between the bottom of the a-wave and the top of the b-wave), as shown in Figure 8. The b-wave value was lower in the hydrogel groups than in the healthy group on days 1, 14 and 28. However, significant differences were not observed between the treated and healthy groups. All results showed that TON could damage retinal function and that the hydrogels exerted limited protective effect on the recovery of retinal function.

Flash visual evoked potential (f-VEP) test was used to assess the functional integrity of the visual pathway from the retina to the visual cortex. As shown in Figure 7C, the N2-P2 wave amplitude of the drug-loaded PPP hydrogel was higher than that in the control, NS and PPP groups. The results provided evidence of the drug-loaded PPP hydrogel treatments preserved vision-related neural activity in the context of traumatic optic nerve injury.

### RGC survival and RGC axonal regeneration *in vivo*

**Figure 9.**
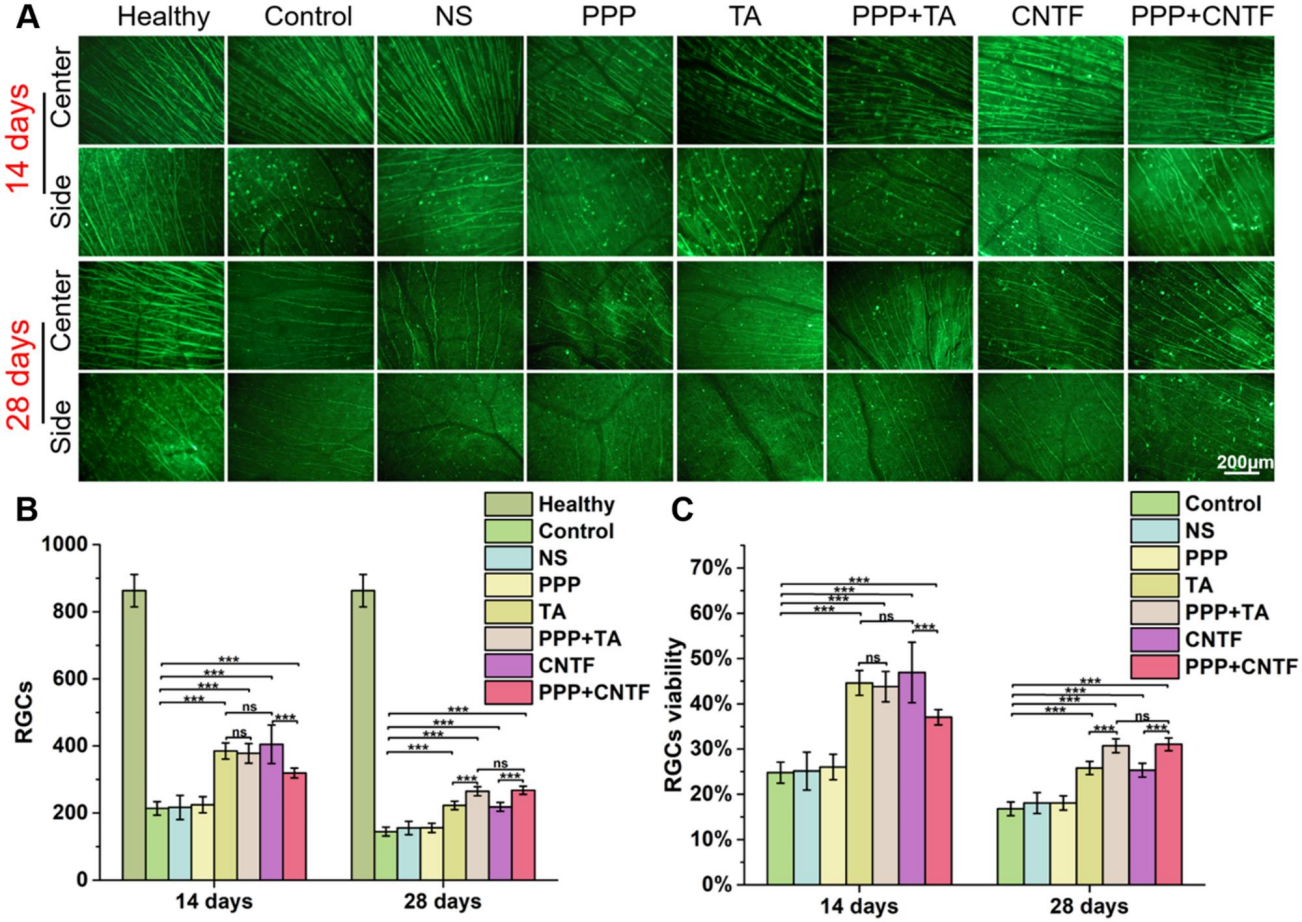
(**A**) Representative micrographs of retinal anti-β-III-tubulin immunostaining of the groups after 14 and 28 days of treatment. (**B**) Quantitation of RGC survival in each group (ns *P* > 0.05, *** *P* < 0.001). (**C**) *In vivo* RGC cell survival rates in each group after 14 and 28 days. Bar: 200 μm.

To assess the effect of the drug-loaded hydrogels on RGC survival in the TON model *in vivo*, we counted the number of RGCs after anti-β-tubulin immunofluorescent staining (Figure 9). At 2 weeks after TON, the retina suffered obvious damage, with 75% loss of RGCs compared with that in the healthy group (Table 2), and the number of RGCs decreased dramatically from 840 ± 52 (healthy group) to 208 ± 17 (control group), indicating successful construction of the *in vivo* TON model. After TON, the number of RGCs decreased in all experimental groups and the axons showed thinner morphology than those of the healthy group. After one single intravitreal injection, a significant increase in the survival of RGCs was observed at 14 days in the TA (44.62 ± 2.75%), PPP + TA (43.81 ± 3.37%), CNTF (46.93 ± 6.67%) and PPP + CNTF (37.05 ± 1.70%) groups compared with that in the control (24.80 ± 2.33%) and NS (25.13 ± 4.19%) groups (*P *<* *0.5). The survival rate of RGCs decreased dramatically at 28 days, with survival rate of 25.81 ± 1.45% for the TA group, 30.74 ± 1.55% for the PPP + TA group, 25.32 ± 1.52% for the CNTF group, and 31.05 ± 1.41% for the PPP + CNTF group, which were still significantly higher than that of control group (16.79 ± 1.50%) and NS group (18.07 ± 2.30%) (*P *<* *0.5). Significant differences in RGC survival were not observed among the PPP, control, and NS groups, demonstrating that the intravitreal injection of PPP hydrogels alone was non-functional to RGC protection, and that this procedure did not cause obvious secondary injury. It is noteworthy that at 28 days, RGC survival in the PPP + TA group was significantly higher than that in the TA group, and that RGC survival in the PPP + CNTF group was higher than that in the CNTF group.

**Table 2.** *In vivo* RGC cell surviving rates (*x* ± *s*)

	Control	NS	PPP	TA	PPP + TA	CNTF	PPP + CNTF
14 days	24.80 ± 2.33%	25.13 ± 4.19%	26.04 ± 2.80%	44.62 ± 2.75%	43.81 ± 3.37%	46.93 ± 6.67%	37.05 ± 1.70%
28 days	16.79 ± 1.50%	18.07 ± 2.30%	18.08 ± 1.59%	25.81 ± 1.45%	30.74 ± 1.55%	25.32 ± 1.52%	31.05 ± 1.41%

### Optic nerve regeneration *in vivo*

**Figure 10.**
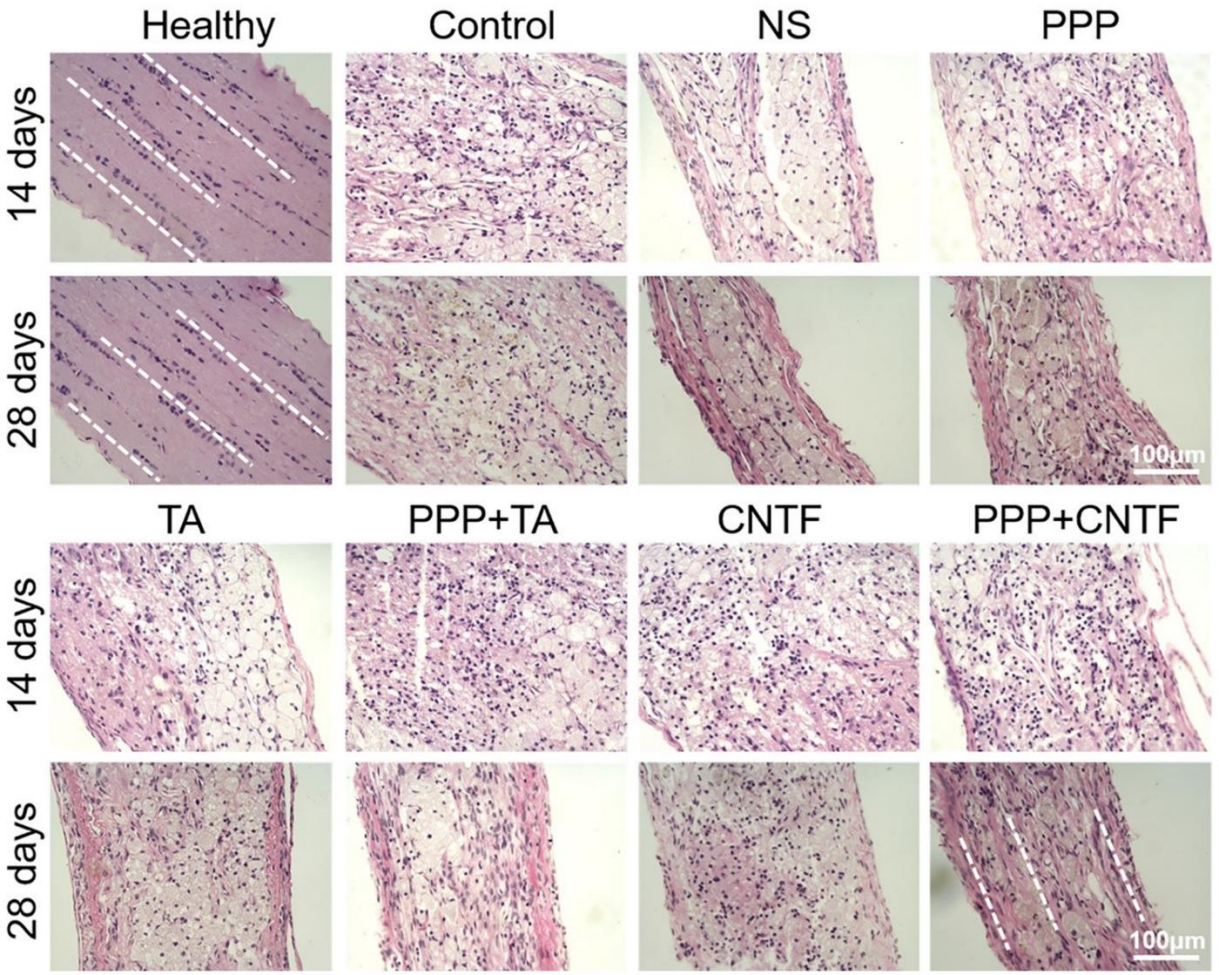
Representative micrographs of optic nerve pathological changes stained using H & E in each experimental group after 14 and 28 days of treatment. Bar: 100 μm.

The optic nerves were carefully exteriorized, longitudinally embedded in paraffin, and sliced for H & E staining after 14 and 28 days to evaluate the *in vivo* inflammatory response of the optic nerve to treatment. The results are presented in Figure 10. The optic nerve of healthy rats showed intact membranes in the outer layer of the optic nerve (dotted white line), and uniformly shaped cells were arranged along the optic nerve (the blue nuclei were along the optic nerve). The orientation of the neural fibres was clear. Fourteen days after TON, obvious inflammation was observed in all groups, along with disordered cell arrangement, non-uniform cell shape, presence of many physaliphorous cells with vacuoles, deep blue nuclei condensation and inflammatory cell infiltration. Pores between the fibres were evident, and the fibres appeared disrupted, twisted and disordered. On Day 28, the fibrous cells proliferated at the injury site of the optic nerve in all TON groups. Inflammatory cell infiltration in the TA, PPP + TA, CNTF and PPP + CNTF groups was less than that in the control group. The TA and PPP + TA groups exhibited the highest levels of inflammatory inhibition.

**Figure 11.**
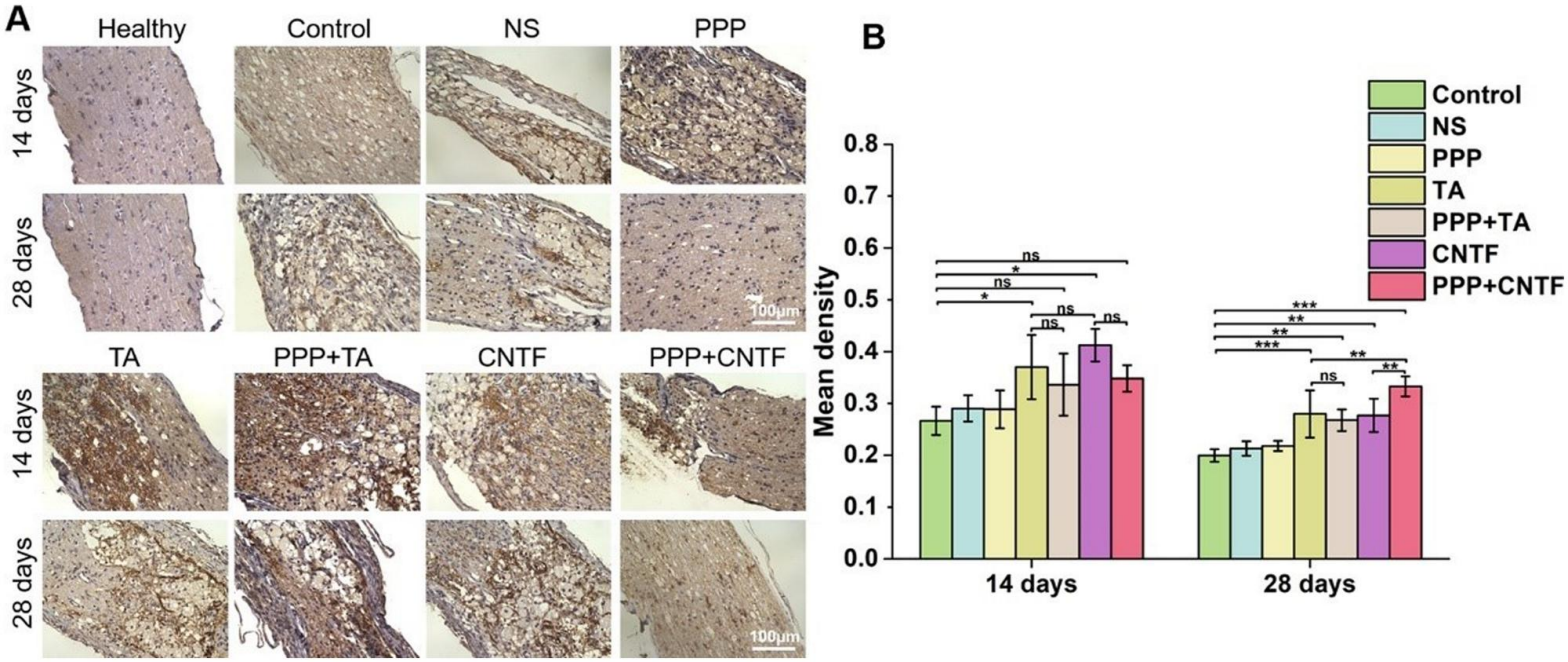
(**A**) Representative longitudinal images of GAP-43 immunostained optic nerve in each group after 14 and 28 days of treatment. (**B**) Quantification of the mean density of optic nerve injury site in each group after 14 and 28 days (ns *P* > 0.05, * *P* < 0.05, ** *P* < 0.01, *** *P* < 0.001). Bar: 100 μm.

GAP-43 immunohistochemical staining of the optic nerve was used to evaluate the protective effects of the intravitreal injection of thermosensitive hydrogels (Figure 11A). A slightly brown colour of positively stained GAP-43 was observed in the healthy group. In contrast, an obvious brown colour because of GAP-43 expression in injured RGCs was observed in the control group. The brown colour was deeper in the TA, PPP + TA, CNTF and PPP + CNTF groups than in the NS, PPP and control groups. The results of quantitative analysis (Figure 11B) showed that at Day 14, the expression of GAP-43 in TA (mean density: 0.37 ± 0.06) and CNTF (0.41 ± 0.03) groups were significantly higher than that in the control group (0.27 ± 0.03) (*P *<* *0.5). Significant differences were not observed between the TA and CNTF groups. The mean densities of the PPP + TA and PPP + CNTF groups were 0.34 ± 0.06 and 0.35 ± 0.03, respectively, which were higher than that of the control group, although not significant. For all groups, the expression of GAP-43 at Day 28 was lower than on Day 14. The mean densities of the TA (0.28 ± 0.05), PPP + TA (0.27 ± 0.02), CNTF (0.28 ± 0.03) and PPP + CNTF (0.33 ± 0.02) groups were significantly higher than that of the control group (*P *<* *0.5). The PPP + CNTF group showed the highest mean density, which was significantly higher than that of the TA and CNTF groups.

### Anti-oxidant and anti-inflammatory effect

**Figure 12.**
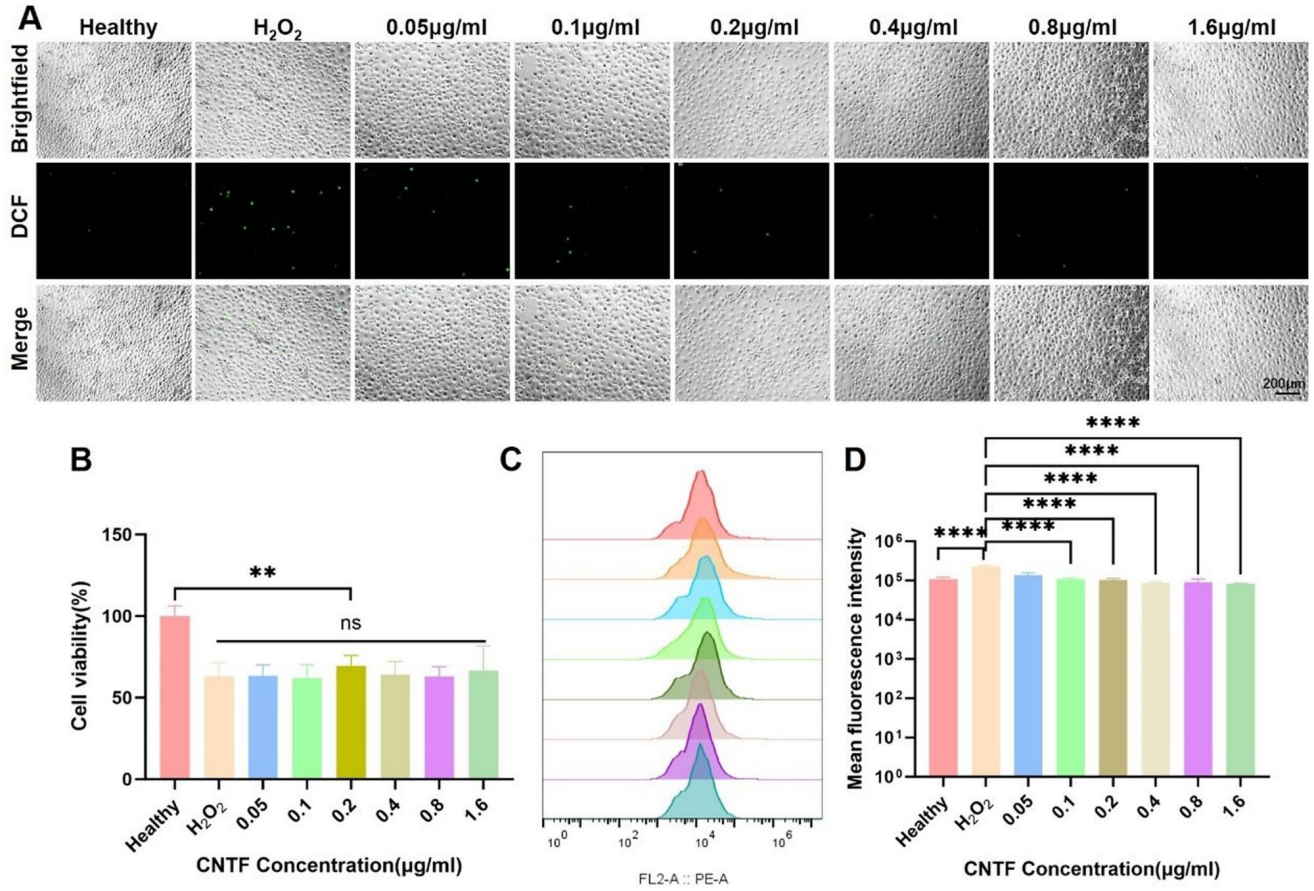
Oxidative stress study of 661 W cells. (**A**) Representative fluorescence images; (**B**) cell viability of 661 W cells at different CNTF concentrations with 300 μM H_2_O_2_; (**C** and **D**) the flow cytometry in the heathy, H_2_O_2_ control and CNTF with different concentration groups, respectively. Data were expressed as mean ± SD. **P <* *0.01, ****P <* *0.0001.

Mouse photoreceptor-derived 661 W cells were used to establish an H_2_O_2_-stimulated oxidative stress model. The induction with H_2_O_2_ caused oxidative damage to 661 W cells in a dose-dependent manner (data not shown). The IC50 was calculated to be 354.4 μM, and therefore, 300 μM H_2_O_2_ was selected to induce oxidative stress in 661 W cells. To evaluate the protective effects of various CNTF treatment on 661 W cell viability, the CCK-8 assay was performed. The results demonstrated that CNTF significantly restored the viability of 661 W cells under oxidative stress (*P* < 0.01), as depicted in Figure 12B. The fluorescence imaging (Figure 12A) and flow cytometer analysis (Figure 12C and D) showed that ROS levels were significantly lower in the CNTF-treated groups compared to the H_2_O_2_-only group (*P* < 0.0001). Additionally, mean fluorescence intensity was reduced in the CNTF groups, indicating a decrease in ROS levels.

**Figure 13.**
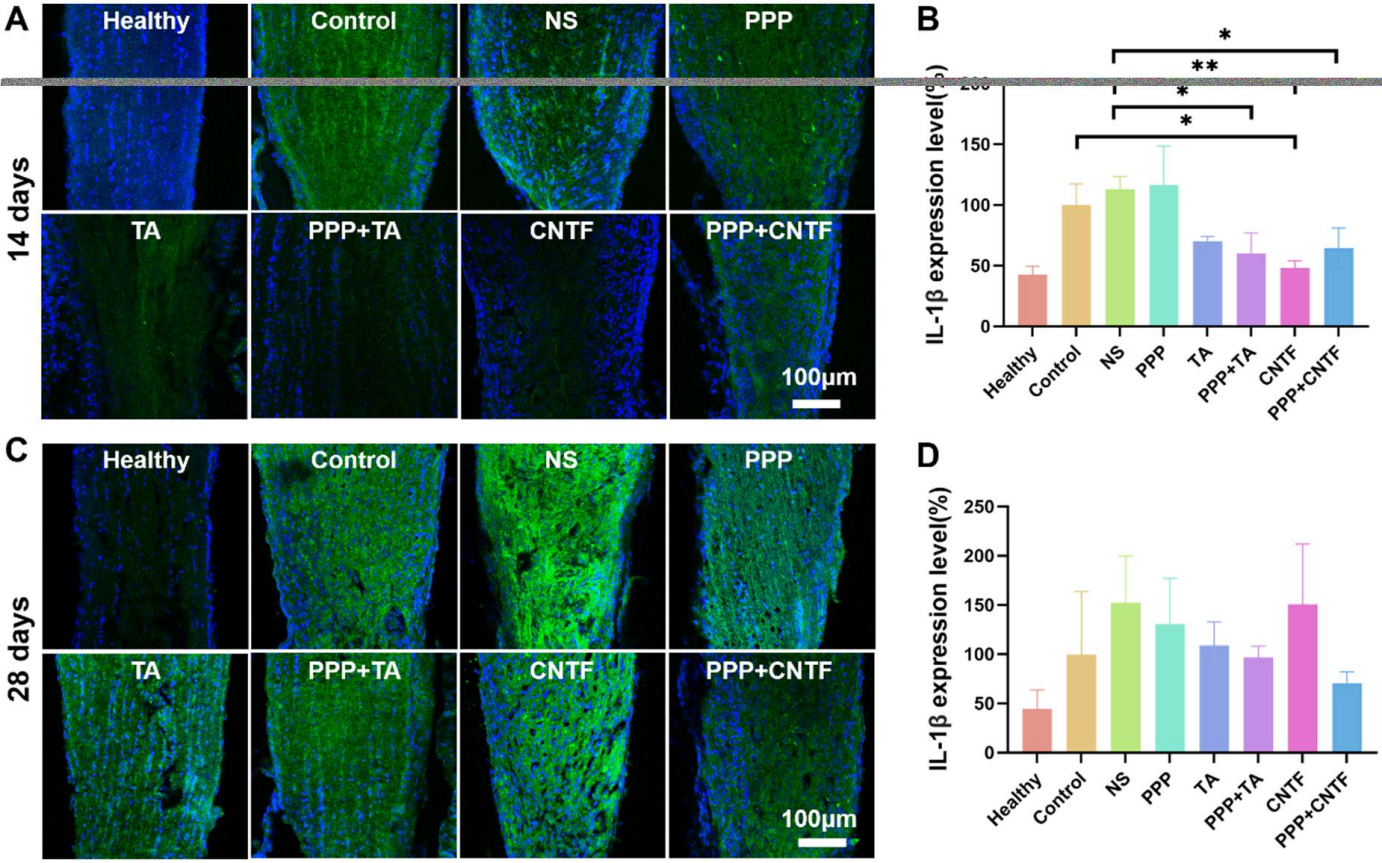
Immunofluorescence result of IL-1β level in optic nerves. (**A** and **C**) the representative images of 14 and 28 days after treatment; (**B** and **D**) the integrated OD of the immunofluorescence of the expression of IL-1β in optic nerves, respectively; data were expressed as mean ± SD. *P < 0.05, **P < 0.01.

IL-1β protein expression level in the optic nerves under different treatments after TON was assessed via immunofluorescence (Figure 13A and C). The consistent quantitative analysis (Figure 13B and D) showed that on Day 14, the expression of IL-1β in PPP+TA, CNTF, and PPP+CNTF groups were significantly lower than that in the control group. On Day 28, the IL-1β expression in the drug-loaded PPP hydrogel groups was lower than that in the NS group without significant differences. For all groups, the expression of IL-1β at Day 14 was lower than at Day 28.

## Discussion

Permanent visual impairment following TON results from the inability of RGC axons to regrow and reform functional connections to their original targets. Loss or defect of vision will impair physical, emotional as well as psychological state of human health. The outcomes of surgical treatments remain controversial [35]. As for medication, there are many barriers, e.g. blood–brain barrier, blood–ocular barriers, cornea–aqueous barriers and intrinsic structure, present challenges in drug delivery to the injured optic nerve lesion. Even delivered to the vitreous body, free drug would be diluted in the vitreous and quickly cleared out of the eye, drug efficacy and durability can be adversely compromised. Thus, there is an imperative need for an RGC-protective drug delivery system for intravitreal injection to avoid these barriers [36]. In our study, a drug-loaded thermosensitive hydrogel has been fabricated to provide stable and effective drug concentration to preserve RGCs after TON. The drug-loaded thermosensitive hydrogel can be injected intravitreally, form a gelled drug reservoir close to retina, release drugs to RGC layer locally and protect the injured RGCs continuously. Our results showed that the thermosensitive hydrogels maintained a stable hydrogel state at body temperature and had the capacity to release drugs in a sustained manner. The drug-loaded hydrogels showed good cytocompatibility, anti-inflammatory and promoted the survival and neurite extension of RGCs *in vitro* and *in vivo*.

In the terms of design and selection of materials, we used PPP based on the following advantages: easy to handle, have amphipathic and amphiphilic chain segment to load hydrophobic drugs and hydrophilic agents, and is temperature-sensitive, showing sol-gel transition near body temperature (Figure 1). Moreover, as a thermosensitive injectable hydrogel, PPP could be applied *in vivo* by a minimally invasive way and bypass intraocular barriers with intravitreal injection [37]. Therefore, PPP was adopted to combine with TA and CNTF separately to study the effect of steroids on inflammation control and the role of growth cytokines in RGC protection and axonal regeneration in TON. Herein, PPP played different roles for different drugs. For CNTF, PPP acted as a drug depot for CNTF to reduce the susceptibility (immunogenicity) and extend its half-life. For TA (a poorly water-soluble steroids), PPP was adopted as a solubilizer to provide instantaneous anti-inflammatory effects via the burst release of TA at first stage and long-term suppression of inflammation via the controlled release of TA at the second stage (Figure 2).

Anatomically, the RGC layer is next to the vitreous body, thus it is necessary to evaluate the *in vitro* biocompatibility of the hydrogel using RGCs, RPE and Müller cells [38]. All the cells were compatible with the extraction of the hydrogel (Figure 3A–C), and the live/dead staining result (Figure 3E) showed consistency. The viability of RGCs in the 300 mg/ml PPP hydrogel group was higher than that of the 250 and 200 mg/ml groups, indicating that extraction of the 300 mg/ml PPP hydrogel was more conducive for the proliferation of RGC cells, which was consistent with the previous study [39]. To sequencing the loading amount of TA, we tested the RPE viability of PPP/TA hydrogel, and the results showed TA between 0.12 and 8 mg per sample was nontoxic. According to the TA release profile (Figure 2A), the 24 h release rate of TA was 12.419%; therefore, the concentration of TA at 24 h was between 0.01242 and 0.8280 mg/ml (the volume of the culture medium was 1.2 ml). This result was not consistent with those of previous studies (TA was directly in contact with the cells) [40], which may be due to the protection provided by the PPP hydrogel against TA [41], indicating that the PPP hydrogel can reduce the contact toxicity of TA.

The *in vivo* safety of the hydrogel was assessed from the fields of toxicity (Figures 6 and 7, slit lamp and OCT) and retinal function (Figure 8, ERG). No obvious inflammatory response was observed in the images, and b-wave amplitude calculated from the ERG diagram decreased after injury in rats with TON compared with that in the healthy group (*P *>* *0.05), albeit not significantly, confirming the safety of the intravitreal injection of the hydrogel. Previous studies showed 25 mg TA injection could induce high intraocular pressure without injuring the optic nerve, which is equal to the concentration of 6.25 mg/ml of TA (the volume of the human vitreum is 4 ml) [40, 42, 43]. For a rat model, the vitreous volume is 56 ± 2 μl, and therefore, the dosage should be less than 0.35 mg. In the present study, we used 0.2 mg TA for the *in vivo* studies. CNTF (0.5 μg) was used as mentioned in previous studies [44–46]. Although some previous studies have shown that chronic inflammation occurs due to the degraded product of PLGA [47, 48], in this study, the results of the slit lamp and OCT analyses showed that intravitreal injection of the PPP hydrogel did not induce observable inflammatory response. This may be due to the microinjection volume (total 5 μl for each injection, no more than 10% of the vitreous volume [49]), indicating that 5 μl was safe for intravitreal injection in rats, which was consistent with the safety range of 3 − 5 μl [27].

In the current study, a mechanical optic nerve crush injury model was adopted due to its (i) ease of operation without craniotomy, (ii) maintenance of the integrity of the optic nerve sheath, (iii) clear injury with minimal trauma, (iv) high postoperative animal survival rate and (v) most importantly, high consistency with the human TON process and its pathophysiology [50]. The RGC survival rate was approximately 25% 14 days after moulding (Figure 9), indicating the successful establishment of the optic nerve injury model (normally 10–30% according to a previous study [51]). Previous studies indicated that RNFL thickness decreases with posttraumatic time [52]. However, our results (Figure 8A–C) showed that the thickness of the RNFL, GCC and entire retina did not change significantly on Days 1 and 14, whereas it decreased significantly by 50% on Day 28. This may be because the reduction in RNFL thickness was hysteretic to RGC damage [53]. Other studies also showed that RNFL thickness did not change in the first 1 week of injury, but decreased significantly after 2 weeks based on OCT observation [54, 55]. With the same method, Higashide *et al.* [56] found that the RGC number decreased by more than 50% 1 week after the injury. These studies also indicated that a reduction in RNFL thickness (axon loss) lagged RGC damage. However, the mechanisms underlying the hysteresis remain unclear. Some researchers attribute this to the interference in the optical properties of the target tissue [57]. In our study, we observed transient white objects fading away over time after the injection therapy. This could interfere with the retinal optical properties and result in hysteresis in the thickness measurement. The results indicated the successful establishment of the TON model.

Primary injury to the optic nerve causes immediate damage from either direct or indirect TON. The subsequent inflammation upregulates apoptosis factors, and the concomitant swelling of optic nerve tissue within the limited bony canal compresses the capillaries to form ischemic damage, resulting in secondary injury due to RGC death. To investigate the *in vivo* protective effects and promotion capacity of the PPP + CNTF and PPP + TA hydrogels, we counted the RGC number in the retina (Figure 9) and quantitatively analyzed the expression of the specific axonal growth marker, GAP-43. The RGC viability and the expression of GAP-43 in the drug-loaded hydrogel groups were significantly higher than that in the control and NS groups on Day 28. These results verified the effectiveness of using intravitreal injection of the drug-loaded hydrogel system. It is noteworthy that the RGC survival rate of the PPP + TA and PPP + CNTF groups were significantly higher than those of the TA and CNTF groups on Day 28, whereas no significant differences were observed on Day 14, indicating that the drug-loaded hydrogel had a long-term protective effect on RGCs. The protection and promotion effect of the drug-loaded hydrogel can be attributed to the solubilization and encapsulation capabilities of the PPP hydrogel on hydrophobic TA and hydrophilic CNTF, separately. Fast-released TA (Figure 2A) was designed to control the inflammatory response in the early stage and regulate post-TON microenvironment to prevent RGCs from rapid deterioration. H&E staining (Figure 10, TA and PPP + TA groups) showed the consistency of the inflammation prevention effect. Our study displayed that both CNTF and PPP + CNTF (Figure 5) groups exhibited increase of the neurite outgrowth when *in vitro* coculturing with RGC. Previous studies showed that the intravitreal injection of CNTF can stimulate retinal gliocytes to express and release endogenous CNTF [58]. We also found that exogenous CNTF directly promoted RGCs axonal growth when co-cultured with the CNTF-combined scaffold (manuscript in preparation). All the results highlighted PPP + TA and PPP + CNTF system can exert better effect of encapsulated drugs than that of pure CNTF and TA, which ensures the potential of drug-loaded hydrogel in the treatment of TON.

To investigate the mechanism of CNTF action in preserving and regenerating the optic nerve, we utilized photoreceptor-derived 661 W cells as a model system. Our findings demonstrated that CNTF exhibits a protective effect on oxidant-treated 661 W cells by significantly reducing ROS levels. This suggests that CNTF may contribute to the preservation and regeneration of optic nerve cells by mitigating oxidative stress-induced damage. Moreover, we found that optic nerve regeneration may also be facilitated by reducing the expression of the cytokine IL-1β. This suggests that the downregulation of IL-1β could play a role in controlling inflammation, which in turn may promote the healing and regeneration of the optic nerve following injury.

## Conclusion

In this study, we have demonstrated that the TA or CNTF incorporated PPP hydrogel is a simple, feasible and efficacious construct for preserving the optic nerve of rats after TON may through anti-oxidant and inti-inflammatory pathways. In contrast to current therapies, such as eye drops and intravitreal injection of pure bioactive agents, PPP + TA and PPP + CNTF is associated with high delivery efficacy and avoidance of pain from frequent injections, and shows high neuroprotective and regenerative capacities. Because of these properties, PPP + TA and PPP + CNTF may provide a new strategy to preserve RGCs. In summary, further development and optimization of drug-loaded thermosensitive hydrogel intravitreal drug delivery system may ultimately lead to a viable treatment for TON.
